# Deciphering the Link Between Hyperhomocysteinemia and Ceramide Metabolism in Alzheimer-Type Neurodegeneration

**DOI:** 10.3389/fneur.2019.00807

**Published:** 2019-07-31

**Authors:** Hervé Le Stunff, Julien Véret, Nadim Kassis, Jessica Denom, Kelly Meneyrol, Jean-Louis Paul, Céline Cruciani-Guglielmacci, Christophe Magnan, Nathalie Janel

**Affiliations:** ^1^Université de Paris, BFA, UMR 8251, CNRS, Paris, France; ^2^Institut des Neurosciences Paris-Saclay (Neuro-PSI), Université Paris-Sud, CNRS UMR 9197, Orsay, France; ^3^AP-HP, Hôpital Européen Georges Pompidou, Service de Biochimie, Paris, France

**Keywords:** NAFLD, Alzheimer's disease, hyperhomocysteinemia, sphingolipid metabolism, ceramides

## Abstract

Aging is one of the strongest risk factor for Alzheimer's disease (AD). However, several data suggest that dyslipidemia can either contribute or serve as co-factors in AD appearance. AD could be examined as a metabolic disorder mediated by peripheral insulin resistance. Insulin resistance is associated with dyslipidemia, which results in increased hepatic ceramide generation. Hepatic steatosis induces pro-inflammatory cytokine activation which is mediated by the increased ceramides production. Ceramides levels increased in cells due to perturbation in sphingolipid metabolism and upregulated expression of enzymes involved in ceramide synthesis. Cytotoxic ceramides and related molecules generated in liver promote insulin resistance, traffic through the circulation due to injury or cell death caused by local liver inflammation, and because of their hydrophobic nature, they can cross the blood-brain barrier and thereby exert neurotoxic responses as reducing insulin signaling and increasing pro-inflammatory cytokines. These abnormalities propagate a cascade of neurodegeneration associated with oxidative stress and ceramide generation, which potentiate brain insulin resistance, apoptosis, myelin degeneration, and neuro-inflammation. Therefore, excess of toxic lipids generated in liver can cause neurodegeneration. Elevated homocysteine level is also a risk factor for AD pathology and is narrowly associated with metabolic diseases and non-alcoholic fatty liver disease. The existence of a homocysteine/ceramides signaling pathway suggests that homocysteine toxicity could be partly mediated by intracellular ceramide accumulation due to stimulation of ceramide synthase. In this article, we briefly examined the role of homocysteine and ceramide metabolism linking metabolic diseases and non-alcoholic fatty liver disease to AD. We therefore analyzed the expression of mainly enzymes implicated in ceramide and sphingolipid metabolism and demonstrated deregulation of *de novo* ceramide biosynthesis and S1P metabolism in liver and brain of hyperhomocysteinemic mice.

## Introduction

Insulin resistance is a major public health outcome by its association with the non-alcoholic fatty liver disease (NAFLD), metabolic syndrome, type 2 diabetes mellitus (T2DM), obesity, and Alzheimer's disease (AD)-type neurodegeneration epidemics. Aging is also a powerful AD risk factor. However, many data imply dyslipidemic conditions as contributor or co-factors in pathogenesis of AD. The classical “amyloid cascade” hypothesis in AD, actually deeply debated, demonstrates that cognitive defects and memory loss implicate the development of wide and insoluble beta amyloid plaques in various brain areas, resulting in apoptosis of neurons (1).

Apart from amyloid-β peptide (Aβ), many evidences suggest that impairing of insulin signaling and brain glucose metabolism act an important role in AD development. Human post-mortem studies support this notion, indicating that insulin resistance in AD brain is systematically showed and increased with disease advancement (2–4). Another connection between T2DM and AD could be supplied by Tau processing failures. Neurofibrillary tangles (NFTs) are principally constituted by hyperphosporylated tau proteins. NFTs, such as amyloid beta plaques, account as important histopathological characteristics of AD. Some authors have called AD as “type 3 diabetes” in relation with the alterations, at the very early disease stages, of insulin signaling more specifically in the brain (2). Insulin resistance is linked with inflammation and dyslipidemia which results in increased ceramides generation notably in hepatic function (5, 6). For this reason, AD could be considered like metabolic diseases mediated by disorders due to peripheral insulin resistance. Progressive hepatic steatosis induces inflammation with activation of pro-inflammatory cytokines, leading not only to increased ceramide production, but also alteration in one carbon metabolism. Ceramides build up in cells because of disruptions in sphingolipid metabolism and activation of pro-ceramides genes (7). Cytotoxic ceramides and related molecules generated in liver promote insulin resistance, traffic through the circulation due to injury or apoptosis caused by local liver inflammation, and because of their hydrophobic nature, can pass through the blood-brain barrier (BBB), thereby exerting toxic responses as reducing insulin signaling and increasing pro-inflammatory cytokines. These defects initiate or support propagation of neurodegeneration with an oxidative stress and ceramide production, exacerbating brain insulin resistance, neuronal apoptosis, and neuro-inflammation. Therefore, neurodegeneration can be caused by toxic liver lipid production (5). In mice, increased production of toxic lipid/ceramide can be induced by liver/peripheral tissue-brain axis of neurodegeneration and be moved through the BBB resulting cognitive impairments (8).

Disturbances in one carbon metabolism, determined by enzyme failures integral to this process, comply with abnormally increased plasma homocysteine (Hcy) levels, namely hyperhomocysteinemia (HHcy). HHcy has been frequently linked with T2DM, cardiovascular diseases (CVD), atherosclerosis and present in NAFLD (9–11). Given that the majority of dietary methionine metabolism is made in liver, this organ represents the major place for Hcy metabolism (12). During liver failure, metabolism of Hcy was modified in association with lipid metabolism disturbance (13–15). Increased Hcy level is also associated with AD pathology (16–19). Taken as a whole, results suggest that Hcy/ceramides signaling pathway exists which suggests a link between Hcy toxicity and intracellular ceramide accumulation via the activation of ceramide synthase (20).

## Homocysteine, a Link Between NAFLD, T2DM, and AD

**Figure 1:**
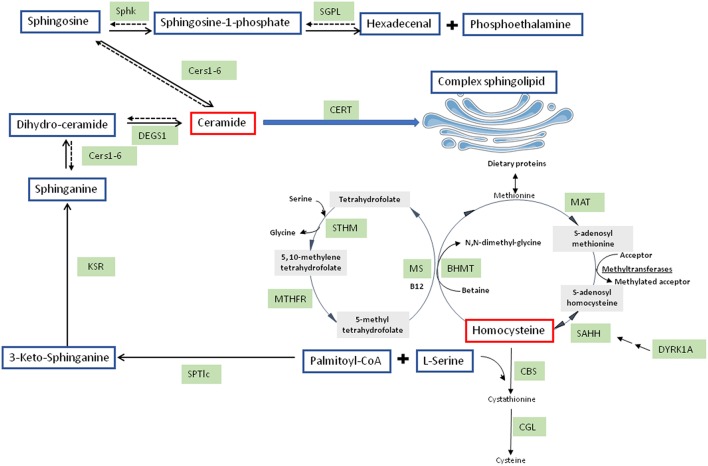
Integrated pathways for homocysteine/cysteine synthesis from methionine and sphingolipid synthesis. BHMT, betaine-homocysteine methyl transferase; CBS, cystathionine beta-synthase; Cers, ceramide synthases; CERT, ceramide transferase; CGL, cystathionine gamma-lyase; DEGS1, dihydroceramide desaturase 1; KSR, 3-keto-sphinganine reductase; MAT, methionine adenosyl transferase; MS, methionine synthase; MTHFR, 5,10-methylene tetrahydrofolate reductase; SAHH, S-adenosyl homocysteine hydrolase; SGPL, sphingosine-1-phosphate lyase; Sphk, sphingosine kinase; SPTlc, serine palmitoyltransferase; STHM, serine transhydroxymethylase.

In the world, NAFLD is the most shared hepatic disorder, its incidence reaching 70–90%. It is also linked with obesity, T2DM and related metabolic diseases (21). NAFLD is linked to hepatic insulin resistance and occurs frequently with obesity/T2DM. Intrahepatic fat accumulation is the feature of NAFLD. Progression of NAFLD is more likely to take place in metabolic diseases patients (22). Oxidative stress associated with insulin resistance have an essential part in NAFLD (23). One-carbon metabolism is involved in methylation of notably proteins, DNA, RNA, and protects cells against oxidation (24). S-adenosylmethionine (SAM) is produced by methionine adenosylation and is the most important methyl-group donor in cellular metabolism (25) (Figure 1). DNA methylation capacity of cells can be modified by a reduction of SAM concentration associated with a reduction in SAM: S-adenosylhomocysteine (SAH) ratio (26). One-carbon metabolism perturbation consequently participates to pathogenesis and NAFLD promotion.

In the liver, Hcy, a thiol-containing amino acid, is involved in metabolism of dietary methionine. SAH metabolism produces Hcy, SAH being produced through methylation reactions implicating SAM and methyltransferases. Hcy can be metabolized by conversion to cysteine via the transsulfuration pathway, the first step involving cystathionine beta synthase (CBS) (27) (Figure 1). In alcoholic fatty liver disease (28) but also in non-alcoholic steatohepatitis (NASH) (29), serum Hcy levels are elevated and good predictor of disease progression. Hepatic steatosis in human patients and in mice with CBS deficiency is associated with HHcy (29–32). In rats, HHcy due to decreased hepatic CBS activity is elicited by NAFLD induced by high fat diet (15). Therefore, NAFLD is early characterized by HHcy. The involvement of dual-specificity tyrosine-(Y)-phosphorylation regulated kinase (DYRK1A) in one carbon metabolism is strengthened by the positive correlation between hepatic protein expression and CBS activity (33, 34). DYRK1A, a protein involved in development, growth and apoptosis (35), is also implicated in Hcy cycle (36, 37) (Figure 1). DYRK1A is also implicated in β-cell mass adjustment and involved in carbohydrate metabolism (38, 39). In mice, DYRK1A is also implicated in liver damage induced by alcohol consumption (34).

Individuals with NAFLD can exhibit neuropsychiatric dysfunction, including anxiety and depression, which frequently precede cognitive impairment and dementia. Factors affecting one-carbon metabolism and consequently elevated Hcy levels have been linked to AD. Divers risk factors of sporadic AD have been characterized by long-term and prospective population and cross-sectional retrospective studies, as hypercholesterolemia, T2DM, and HHcy (40). Elevated proinflammatory cytokines have metabolic connotation (40). Older patients suffering from mild hypertension showed association between hippocampal atrophy, and white matter atrophy, with HHcy. Elderly patients with HHcy showed increased rate of hippocampal atrophy and cognitive decline (41, 42). In the aged population, elevated Hcy level can be established as a risk factor for cognitive decline (43). Progression of white matter hyperintensities and faster rates of total brain volume loss have been associated with increased plasma Hcy levels in patients with hypertension (19). Many studies have demonstrated that moderately elevated Hcy levels increased late-onset Alzheimer's disease (LOAD) risk, even if the value is close to the critical threshold (16–19, 44). According to recent international consensus statement (45), moderately elevated Hcy level can increase the relative risk of dementia in the elderly 1.15- to 2.5-fold, with the Population Attributable risk from 4.3 to 31%.

Many mechanisms have been suggested to connect elevated Hcy level with AD. Many experimental studies have demonstrated that elevated Hcy level can produce many neurotoxic effects implying excitotoxicity, oxidative stress, mitochondrial dysfunction, DNA damage and apoptosis. Therefore, HHcy can participate to AD neurodegeneration (46). Preclinical studies show that HHcy generates Aβ accumulation in brain (47–51) and increased hyperphosphorylation of tau (52). The link of Hcy to Aβ can lead to the development of interconnections and consequently to the development of aggregates (53). Elevation of Hcy contributes to the decrease of SAM levels. Demethylation of DNA can be produced by decreased SAM level, leading to overexpression of presenilin (PSEN1) and beta-secretase (BACE1), the β-site amyloid precursor protein (APP)-cleaving enzyme (54). Another study demonstrates that dimerization of apoE3 can be block by Hcy, reducing apoE3-mediated high-density lipoprotein (HDL) generation and consequently reducing microglial degradation of soluble Aβ (55). Patients with HHcy, compared to control subjects, have lower ratio of apoE3 dimers in their cerebrospinal fluid (CSF).

Many studies demonstrate the involvement of DYRK1A in AD (56). DYRK1A interacts with APP, and plays a role in APP processing by direct phosphorylation of APP at Thr-668 and indirect phosphorylation of PSEN1 at Thr-354, promoting the pathological Aβ pathway and the production of Aβ. Increased DYRK1A expression enhances APP phosphorylation and its cleavage, resulting in increased Aβ40 and Aβ42 levels and thus promoting brain β-amyloidosis (57). Its direct tau hyperphophorylation and indirect phosphorylation of alternative splicing factor promotes neurofibrillary degeneration, showing its involvement in neurodegenerative processes and neuronal depletion appearing in AD (57–60). We have previously shown, on the one hand, that plasma DYRK1A levels correlate positively with CSF tau and phosphorylated-tau proteins in AD (61), and on the other hand that combined assessment of plasma levels of DYRK1A and Hcy can be validate as diagnostic marker for AD (44). Lipidomics analysis showed that not only triglycerides (TG) content but also ceramide content were increased in HHcy mice which have decreased liver DYRK1A protein expression (36, 62). We previously demonstrated altered lipoprotein metabolism in mice overexpressing DYRK1A (63). Note that mice overexpressing DYRK1A have, on the contrary, not only a decreased plasma Hcy level (37) but also a decreased serum TG level (0.369 ± 0.04 vs. 0.519 ± 0.057 mmol/L; *p* < 0.05 by Student's *t*-test; *n* = 8 for each). It will be important to demonstrate if the effects of Hcy not only in liver but also in brain are mediated by ceramide signaling.

## Sphingolipid Metabolism and AD

Considered for a long time as structural compounds, several evidences demonstrated that bioactive sphingolipids are involved as signaling molecules in the various tissues including the brain. In these tissues, sphingolipids play important role in several pathologies including neurodegenerative diseases, such as AD. Sphingolipids could be produced by several pathways. *De novo* sphingolipid synthesis is initiated in the cytoplasmic face of the endoplasmic reticulum (ER) which is started with the condensation of palmitoyl-CoA L-serine with to form 3-ketosphinganine (Figure 1). Serine palmitoyl-transferase (SPT) catalyzes this reaction (64). Two subunits, SPTLC1 and SPTLC2, showing a similarity at amino acid sequence of around 20%, compose the heterodimer SPT. SPTLC1 and SPTLC2 seem to be both required for enzyme activity. However, the SPTLC2 subunit contains a pyridoxal phosphate binding motif (65) and produces the common and major C18-sphingoid bases. A third SPT subunit, SPTLC3, has been identified, with 68% homology to the SPTLC2 subunit. However, SPTLC3 is involved in the production of C14- and C16-sphingoid bases. SPTLC2 and SPTLC3 are therefore distinct from a specificity point of view (66). The SPT substrate preference toward the requirement of longer acyl-CoA could be modulated by the differential expression of SPTLC2 and SPTLC3. Moreover, C16-sphingoid bases could be transformed into more complex sphingolipids, such as C16-ceramide and glycosphingolipid. Sphingolipids can be implicated in AβPP/Aβ metabolism and therefore AD development due to their structural roles in cellular membranes including lipid rafts (67). Conversely, ceramide formation can be promoted by accumulation of oligomerized Aβ in AD brain. Indeed, Aβ peptides can activate SPT, resulting in neurotoxic ceramide increase by the *de novo* synthesis pathway (68, 69). Interestingly, SPTLC2 was found to be up-regulated in AD (70, 71). However, a regulation of SPTLC3 subunit has not been explored in neurodegenerative diseases development, such as AD.

3-ketosphinganine is formed and rapidly reduced by 3-ketosphinganine reductase into dihydrosphingosine (DH-Sph) (Figure 1). The reaction results in DH-Sph, implicated in the production of various species of dihydro-ceramides by ceramide synthases (CerS) (Figure 1). This species will differ by the nature of acyl-CoA chain length used for the N-acylation of DH-Sph (72). The dihydroceramide desaturases (DEGS1) metabolizes dihydro-ceramides into ceramides (Figure 1). Interestingly, CerS1 and CerS2 are up-regulated in AD brains whereas CerS6 is reduced (70, 71), suggesting a remodeling of ceramide species during the development of AD. The ceramide produced are converted in the Golgi apparatus into sphingomyelin or glucosyl-ceramides by sphingomyelin synthase and glucosyl-ceramide synthase, respectively (73) (Figure 1).

Ceramidases could deacylated ceramides to produce sphingosine (Figure 1). In cells, sphingosine but also other sphingoid bases can be phosphorylated by two sphingosine kinases (SphK 1 and SphK2) to form sphingosine-1-phopshate (S1P). S1P can be dephosphorylated to sphingosine by specific S1P phosphohydrolases, the reaction being reversible, or can be cleaved by a pyridoxal-dependent S1P lyase (SGPL) into ethanolamine phosphate and hexadecenal, the reaction being irreversible (74) (Figure 1). S1P, in contrary to ceramide, is known to be a pro-survival lipid for various cells including neurons (74). Interestingly, S1P metabolism has also been related to AD. Indeed, FTY720, a substrate of SphK2 that can bind S1P receptors has been shown to reduce neuronal Aβ generation (75). Loss of neuroprotective S1P and SPHK activity was found early in AD development prior to AD diagnosis with a decrease of Sphk2 activity in hippocampus (76). However, the role of SphK2 is still controversial in AD since S1P production by SphK2 and Aβ processing seemed to be positively correlated (77). This discrepancy could come from subcellular distribution of SphK2 between cytosol and nucleus which is altered in AD brains (78). These data suggest that the pro-survival cytosolic S1P may be less efficient by a shift in the subcellular localization of the S1P generating by SphK2 which will lead to the production of nuclear S1P associated with deleterious effects in AD pathogenesis. Up to date, SphK2 and SGPL (70) have been shown to be overexpressed whereas SphK1 was found to be down-regulated in AD brain suggesting a deregulation of S1P signaling in this pathology which remained to be clarified.

## Ceramides, a Link Between NAFLD, T2DM, and AD

Ceramides are also important mediators of insulin resistance in various peripheral tissues but also in pancreatic β cell deregulation induced by obesity (79, 80). Obesity is well-established as a predisposing factor for the appearance of hepatic steatosis, NAFLD being strongly associated with both hepatic and peripheral insulin resistance with a defect in the ability of insulin to suppress endogenous glucose production (81). In addition, NAFLD is linked to important hepatic changes in lipid metabolites, such as an increase in hepatic cholesterol. When excess of saturated fatty acids are poorly incorporated into hepatocyte triglycerides, they induce lipotoxicity, resulting in liver injuries (82). Excess of saturated fatty acids constitutes a preferential substrate for the *de novo* ceramide biosynthesis (83). It is known that ceramide levels contribute to the development of NAFLD by mediating obesity, inflammation, insulin resistance, and oxidative stress (84).

In addition to intracellular-based actions, circulating extracellular ceramides have roles in insulin resistance. *In vitro* studies showed that hepatocytes treated with palmitate could efficiently secrete newly synthesized ceramide in response to hyperlipidemia, demonstrated by increased extracellular ceramide concentration (85). Ceramide could be either transported by lipoproteins but also by cell-derived membrane shed both basally and under stress conditions called extracellular vesicles (86, 87). Interestingly, advanced pathological signs of AD and also induced neuronal apoptosis could be elicited by chronic NAFLD in mice (88). Therefore, chronic inflammation induced by obesity-associated NAFLD outside from the brain is sufficient to induce neurodegeneration in the absence of genetic predisposition. Since NAFLD is associated with an increase of circulating sphingolipid, such as ceramide, it is tempting to suggest that circulating ceramide originating from liver could also target brain tissues and favor the development of AD.

Some of sphingolipids could constitute biomarkers to identify individuals who are at risk to develop T2DM. Therefore, it will be more important to quantify circulating sphingolipid concentrations. Plasma dihydro-ceramides levels were showed effectively to be significantly elevated up to 9 years before the detection of the disease of individuals from two human cohorts who will progress to diabetes (89). Interestingly, deregulation of ceramide metabolism reflected by an increase of plasma ceramide level could arise in different stages of AD progression (90, 91). Moreover, it would be important to define whether AD is associated with a differential distribution of ceramides in lipoproteins, but also in exosomes, which could serve as biomarkers of disease progression.

S1P has been also shown as a potent regulator of NAFLD, treatment with S1P enhancing hepatic lipid storage (92). A 2-fold increase of SphK1 was determined in livers from humans with NAFLD but also in mice feeded with a high saturated fat diet (92, 93). These mice showed activation of NFκB, elevated cytokine production, and immune cell infiltration. Importantly, a total SphK1-null mice were protected from these outcomes. To date, the role of SphK2 has not been explored in the context of NALFD. In contrast, it has been shown that a total SPL-null mice resulted in a widespread change in lipid metabolism genes expression pattern, with a significant increase in the expression of PPARgamma, a key transcriptional regulator of lipid metabolism (94), suggesting that regulation of SGPL could be a potent regulator of NAFLD. The liver is known to be engaged in regulating the plasma level of S1P, as liver produces a chaperone for S1P transport, the apolipoprotein M (apoM) (95). Interestingly, polarized endothelial cells, composing the lining of the BBB, also express and secrete apoM toward the brain as well as to the circulation suggesting that circulating S1P could target the brain by this way (96). Therefore, as ceramide, secretion of S1P by hepatocytes could constitute a potent regulator of AD by targeting the brain.

## Statement of Hypothesis: Ceramide and Sphingolipid Metabolism is Modified in Liver and Hypothalamus of Hyperhomocysteinemic Mice on a High Fat Diet

Based on the results described above, we used mice heterozygous for targeted disruption of the Cbs gene (Cbs^+/−^) (30) and wild type (Cbs^+/+^) mice on the same background, fed on a standard diet supplemented with 0.5% L-methionine (Sigma-Aldrich, France) in drinking water to induce intermediate HHcy in Cbs^+/−^ mice (97), and with a high-fat diabetogenic diet (HFD) (98). As expected, Cbs^+/−^ mice showed a significant increase of plasma Hcy level (37.1 ± 3.2 μM vs. 6.4 ± 0.4 μM; *p* < 0.0001 by Student's *t*-test *n* = 8 for each). We previously evaluated plasma Hcy levels in transgenic mouse models of AD. No significant difference was observed in blood of transgenic mouse models of AD compared to control mice, indicating that Hcy is not a primary cause of AD (99). Animal studies using AD-like transgenic mouse models, on a methionine enriched diet, were used to provide potential mechanisms by which HHcy might influence AD development. Tg2576 transgenic female mice expressing hAPP with the Swedish mutation (K670N/M671L) on a methionine enriched diet exhibited an increase from 6 to 35 μM (51, 100).

### Effect of HHcy and HFD on Enzymes Expression Involved in Ceramide and Sphingolipid Metabolism in Liver

**Figure 2:**
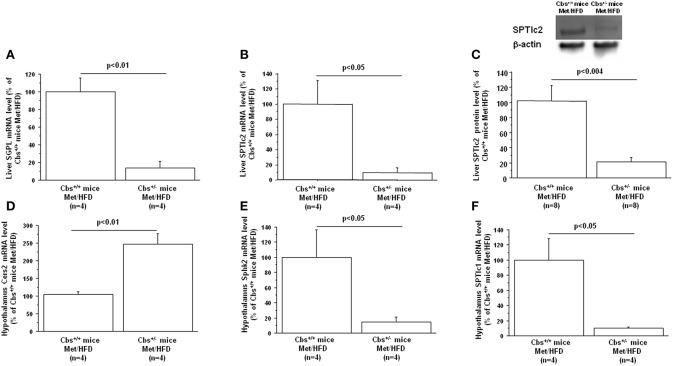
Effect of hyperhomocysteinemia and high fat diet on liver **(A)** SGPL1 mRNA, **(B)** SPTlc2 mRNA, **(C)** SPTlc2 protein, **(D)** Cers2 hypothalamus mRNA, **(E)** Sphk2 hypothalamus mRNA, and **(F)** SPTlc1 hypothalamus mRNA level in wild type (Cbs^+/+^ mice) and mice heterozygous for targeted disruption of the Cbs (Cbs^+/−^ mice) on a methionine enriched diet (Met) and high fat diet (HFD). Relative liver and hypothalamus mRNA expression was described in Table 1. Liver SPTlc2 was determined by western blot and quantified by slot blotting [1/1,000 (101)]. β-actin (1/10,000) (Sigma-Aldrich, France) was used as an internal control for Western blot analysis or ponceau-S coloration for slot blot analysis. Data are presented as mean ± SEM, and analyzed with the Student's *t*-test by using Statview software. *n*, number of mice. Data were considered significant when *p* < 0.05.

Gene expression of mainly enzymes implicated in ceramide and sphingolipid metabolism has first been analyzed by Q-PCR in mice liver. As expected, Cbs^+/−^ mice showed a significant decrease of liver CBS activity (45.2 ± 11.1 vs. 102 ± 21.6; *p* < 0.05 by Student's *t*-test *n* = 4 for each), commensurate with a decrease in mRNA expression (13.5 ± 7.3 vs. 100 ± 26; *p* < 0.03 by Student's *t*-test *n* = 4 for each). Cbs^+/−^ mice also showed a significant decrease of liver Dyrk1A level (40.6 ± 6.3 vs. 100 ± 16; *p* < 0.02 by Student's *t*-test *n* = 4 for each) (36), associated with a decrease in mRNA expression (18.9 ± 14 vs. 100 ± 21.3; *p* < 0.023 by Student's *t*-test *n* = 4 for each). SGPL1 and SPTlc2 expression were decreased in liver of Cbs^+/−^ on methionine and HFD (Table 1) (Figures 2A,B). In agreement with the decreased gene expression of SPTlc2, we also found a decrease in protein level (Figure 2C). Hepatic expression of CBS and SGPL1 (*r* = 0.96, *p* < 0.02), Sphk2 (*r* = 0.83, *p* < 0.05), and SPTlc2 (*r* = 0.93, *p* < 0.03) were positively correlated, Dyrk1A and Cers5 expression being correlated negatively (*r* = −0.93, *p* < 0.02).

**Table 1:** Relative liver and hypothalamus mRNA expression based upon Q-PCR data obtained from wild-type (Cbs^+/+^ mice) and mice heterozygous for targeted disruption of the Cbs (Cbs^+/−^ mice) on a methionine enriched diet (Met) and high fat diet (HFD).

**mRNA (%)**	**Hypothalamus Cbs^**+/+**^ mice Met/HFD (*n* = 4)**	**Hypothalamus Cbs^**+/−**^ mice Met/HFD (*n* = 4)**	**Liver Cbs^**+/+**^ mice Met/HFD (*n* = 4)**	**Liver Cbs^**+/−**^ mice Met/HFD (*n* = 4)**
Cers1	100 ± 52.2	354 ± 211	100 ± 71	107.7 ± 52.8
Cers2	104 ± 8	247 ± 29^**^	100 ± 21	124 ± 15
Cers3	100 ± 26	115 ± 48	100 ± 41	95 ± 24
Cers4	99.9 ± 63.9	103.1 ± 58.8	100.9 ± 29.3	101.7 ± 55.1
Cers5	100 ± 18	226 ± 87	100 ± 28	189 ± 43
Cers6	100 ± 33	157 ± 58	100 ± 30	82 ± 21
CERT	100.4 ± 22.5	40.8 ± 17.1	100 ± 35.2	48.2 ± 25.4
DEGS1	100 ± 38.9	61.1 ± 26.1	100 ± 10.3	105.8 ± 29.6
SGPL1	100 ± 51.6	57.3 ± 23	100 ± 15.3	13.5 ± 7.4^**^
Sphk1	99.3 ± 27.9	71.6 ± 39.2	100.4 ± 44	21 ± 10
Sphk2	100.1 ± 36.4	14.8 ± 6.5^*^	100.4 ± 24.2	45 ± 21.6
SPTIc1	100 ± 28	10.5 ± 1^*^	100 ± 22.9	115.4 ± 32.3
SPTIc2	100 ± 2	52 ± 20.6	100 ± 31.6	9.5 ± 6.3^*^
SPTIc3	98.3 ± 47	371.3 ± 118.3	97.5 ± 24.9	294.5 ± 144.6

*Total RNA was isolated from the hypothalamus and liver, reverse transcribed and real time quantitative PCR amplification reactions were carried as described using the LightCycler FastStart DNA Master plus SYBR Green I kit (Roche) (97). The mRNA transcript level was normalized against the mean of two genes: H1a and TBP (Table S1). Data were normalized to the mean of Cbs^+/+^ mice on Met/HFD. Data are presented as mean ± SEM, and analyzed with the Student's t-test by using Statview software. n, number of mice*.

* p < 0.05;

** *p < 0.01. Data were considered significant when p < 0.05*.

Ceramide is a potent regulator of cell proliferation, activation, and apoptosis. Ceramide plays an key role in different cellular functions, such as plasma lipoprotein metabolism and cell membrane formation, known to contribute to the development of atherosclerosis and other sclerotic diseases, such as insulin resistance, obesity, and AD (102, 103). Previous results have demonstrated that *de novo* ceramide biosynthesis is implicated in induction of kidney NAD(P)H oxidase activity in HHcy rats fed a folate-free diet and report the important role of redox signaling catalyzed by ceramides in glomerular injury induced by HHcy in rats (104). Acid sphingomyelinase is also involved in the development of glomerular oxidative stress and injury induced by HHcy (105, 106).

In HHcy induced by supplementation of Hcy in drinking water for 6 weeks in mice, hepatic steatosis was found to be associated with a notable increase in ceramide-related metabolites and subsequent upregulation of ceramide synthesis genes including *Sptlc3, Degs2, Cer4*, and *Smpd4* (62). Moreover, ceramide synthases were suggested to be involved in Hcy-induced ceramide production by the fact that abolishing the expression of *Sptlc3* and *Degs2* by omega-3 significantly ameliorated HHcy-mediated increases of hepatic ceramide (62). In our study, the increase of Cers5 (correlating negatively with Dyrk1A expression), the non-significant increase of *Sptlc3* support an increased ceramide levels in liver of Cbs^+/−^ mice on methionine and HFD mediated through specific ceramide synthases. The SPTLC3 subunit has been identified as generating short chain sphingoid bases (66) compared to SPTLC2. Commensurate with the increased *Sptlc3* level, *Sptlc2* was found to be decreased in liver of Cbs^+/−^ mice on methionine and HFD, correlating with CBS expression. Palmitate-CoA is the predominant substrate for SPTLC2, whereas mysristoyl and lauryl-CoA is the preferential substrates for SPTLC3, which results in the production of different chain length of the sphingoid base. It is possible that these two subunits can be switched in a SPT enzyme complex to replenish the ceramide pool, knocking down all SPTLC subunits being necessary to decrease total ceramides significantly (107). The hepatic decrease of SPTLC2 in Cbs^+/−^ mice on methionine and HFD could therefore lead to a decrease of ceramides with a C18-sphingoid bases backbone and promotes the synthesis of ceramide species through the action of SPLTC3 with a C16-sphingoid base backbone. Interestingly, SPTLC3 expression has been associated with NAFLD (108) and therefore could participate to its development under the context of HHcy. We also found a strong hepatic decrease of SGPL in Cbs^+/−^ on methionine and HFD suggesting an altered catabolism of S1P. Knowing the novel role of S1P in hepatic injury, such as NAFLD (109), it will tempting to propose that S1P metabolism also could contribute to NAFLD in Cbs^+/−^ mice on methionine and HFD (109). More importantly, down-regulation of SGPL could also contribute to increased circulating S1P levels since it has been shown that saturated fatty acids serve to the synthesis of S1P in hepatocytes which is released in the extracellular environment (110). Increasing S1P levels in HHcy mice could have repercussion on glucose homeostasis by targeting peripheral tissues but could also target the brain where S1P is a potent regulator of AD development (111). Hypothalamic insulin resistance and lipotoxicity have been previously reported to be induce by *de novo* ceramide biosynthesis (101). Therefore, we also analyzed the main enzymes implicated in ceramide and sphingolipid metabolism in hypothalamus of mice.

### Effect of HHcy and HFD on Enzymes Expression Involved in Ceramide and Sphingolipid Metabolism in Hypothalamus

As expected, Cbs^+/−^ mice showed a significant decrease of CBS mRNA expression in hypothalamus (15 ± 10 vs. 100 ± 29; *p* < 0.03 by Student's *t*-test *n* = 4 for each). A significant increase of hypothalamus Dyrk1A level (259 ± 22.3 vs. 101.4 ± 25.2; *p* < 0.003 by Student's *t*-test *n* = 4 for each) was found as expected, without difference in mRNA level (58.9 ± 21.1 vs. 100 ± 30.7; *n* = 4 for each) (112). Expression of Cers2 was increased, with a decrease of Sphk2 and SPTlc1 in hypothalamus of Cbs^+/−^ mice on methionine and HFD (Table 1) (Figures 2D–F). A positive correlation was found between hypothalamic expression of CBS and SGPL1 (*r* = 0.76, *p* < 0.04), Sphk2 (*r* = 0.81, *p* < 0.03), and SPTlc1 (*r* = 0.94, *p* < 0.04). We also found a positive correlation between liver expression of CBS and hypothalamic expression of Sphk2 (*r* = 0.86, *p* < 0.02), and SPTlc1 (*r* = 0.79, *p* < 0.04).

It has been demonstrated that Hcy-treatment of cerebral endothelial cells induces acid sphingomyelinase ceramide pathway (113). The increase of *Cers2*, the decrease of *Sphk2* with the non-significant increase of *Sptlc3* and the non-significant decrease of *SGPL* (*Sphk2 and SGPL* correlating positively with CBS expression) support increased hypothalamic ceramide levels in HHcy mice on HFD mediated through the regulation of specific ceramide synthases. Previous results found in human post-mortem brain and mouse transgenic AD model an increased mRNA level of *Cers1*, Cers2, and a decrease in Sphk1 and Sphk2 (68, 76, 114). Loss of neuroprotective S1P and SPHK activity was found early in AD pathogenesis prior to AD diagnosis (76). Altogether, our results suggest that local alteration of S1P metabolism also could contribute to AD development associated with HHcy.

## Conclusion

In this study, we used HHcy mice due to CBS deficiency to analyze expression of the main enzymes implicated in ceramide and sphingolipid metabolism. Our results support an increased ceramide levels in liver of HHcy mice, particularly implicated in NAFLD, and altered hepatic catabolism of S1P, which could target the brain where S1P is a potent regulator of AD development. Our results also support an increased hypothalamic ceramide levels in HHcy mice, with a local alteration of S1P metabolism. Altogether, our study emphasizes the role of Hcy/ceramides pathway in AD pathology.

## Ethics Statement

All procedures were carried out in accordance with the ethical standards of French and European regulations (European Communities Council Directive, 86/609/EEC). Official authorization from the French Ministry of Agriculture was granted to perform research and experiments on animals (authorization number 75-369) and the experimental protocol was approved by the Institutional Animal Care and Use Committee of the Paris Diderot University (CEEA40), and the agreement # 8728 was given to the project.

## Author Contributions

HL and NJ made the review of the literature and wrote the manuscript. HL, CC-G, CM, and NJ designed the study. JV, NK, JD, KM, J-LP, and CC-G performed the experiments. All authors read and approved the manuscript.

### Conflict of Interest Statement

The authors declare that the research was conducted in the absence of any commercial or financial relationships that could be construed as a potential conflict of interest.
