# Supplementary material for: Deciphering the Link Between Hyperhomocysteinemia and Ceramide Metabolism in Alzheimer-Type Neurodegeneration
Source: Front Neurol. 2019 Jul 31;10:807. doi: 10.3389/fneur.2019.00807 (PMC6684947; doi:10.3389/fneur.2019.00807)
Supplement: Supplementary file 1 [file Data_Sheet_1.docx]

**Table S1**. Quantitative PCR primer sequences

| Gene | Sequence (5'->3') |
| --- | --- |
| Cers1 S | GGAGCTACTGCGCTTACCTG |
| Cers1 AS | CCATGCCTGACCTCCAGT |
| Cers2 S | CCGAGATGCTCCAGACCTTGTA |
| Cers2 AS | GGCATAGACACGTCCATCTTTGTCTTCTA |
| Cers3 S | GGCGATTTACATTTTACTTGCTG |
| Cers3 AS | TCATATGCCCATGGTTTGTC |
| Cers4 S | TTCCCAGTGGCTCTGGTC |
| Cers4 AS | GGCAAGGCCACAAATCTCT |
| CErs5 S | CATGCCATCTGGTCCTACCT |
| Cers5 AS | GCGGTCATCCTTAGACACCT |
| Cers6 S | GGAGCTGTCATTTTATTGGTCTTT |
| Cers6 AS | GGAACATAATGCCGAAGTCC |
| CERT S | TCGCAATGACTGGGAAACTA |
| CERT AS | AAACGATGATTGCATTATCAGC |
| DEGS1 S | GGCTATCATAACGAGCACCAT |
| DEGS1 AS | CACTTGCGATCTTCCTCACC |
| Dyrk1A S | TCAGTCTTCAGGCACCACCT |
| Dyrk1A AS | TGTTACTCGTTCCCGAGGAT |
| H1a S | AGAAGAACAACAGCCGCATC |
| H1a AS | TGCACCAGTGTGCCTTTATT |
| SGPL1 S | GGATGACTTGTTCCCTCTTCA |
| SGPL1 AS | TTTCCGTTCCCCCAGAAG |
| SphK1 S | TGCATCTGGAAGCTAAAAATCTG |
| SphK1 AS | GAGGGTGTCTGGCGACTG |
| SphK2 S | AGGAAACTCGGCCAGACAC |
| SphK2 AS | TCCTAGGCTCTTGGCTTGTG |
| SPTlc1 S | GGTGCTGGTGGAGATGGT |
| SPTlc1 AS | GGATTCCTTCCAAAATAAGATGG |
| SPTlc2 S | TCGGTGCTTCAGGAGGATAC |
| SPTlc2 AS | GAGAATGTGTGCGCAGGTAG |
| SPTlc3 S | GCAAAGCACTGGTTGTGTGA |
| SPTlc3 AS | TCCTCAGCATATGCCTTCCT |
| TBP S | GGGGAGCTGTGATGTGAAGT |
| TBP AS | CCAGGAAATAATTCTGGCTCA |
